# Spontaneous complete migration of suture material after subcuticular continuous suture in cesarean section: a case report

**DOI:** 10.1186/1471-2482-14-103

**Published:** 2014-12-06

**Authors:** Ki-Jin Ryu, Ki Hoon Ahn, Soon Cheol Hong

**Affiliations:** Department of Obstetrics and Gynecology, College of Medicine, Korea University and Korea University Medical Center, 126-1, 5-Ga Anam-Dong, SeongBuk-Gu, Seoul, 136-705 Republic of Korea

**Keywords:** Suture material migration, Subcuticular suture, Wound recovery, Cesarean section

## Abstract

**Background:**

Skin closure during cesarean section is often performed with subcuticular running sutures by using a nonabsorbable suture material. However, this material has the risk of incomplete removal after wound healing and can migrate to other sites in rare cases.

**Case presentation:**

A 34-year-old Korean woman who had undergone a cesarean section 5 months prior presented with a fine, blue object visible through the skin on her left lower abdomen. No pain or any other signs of inflammation were observed. The foreign body was revealed to be 10-cm-long suture material that had migrated laterally approximately 15 cm in intradermal layer during the previous 5 months, without tangling of the entire length.

**Conclusions:**

Small remnants of suture materials in the subcutaneous tissue are known to migrate toward the superficial layer. The mechanism of these migrations is often thought to be related to foreign body immune reaction or the force generated in wound contracture. Long-distance migration of relatively long suture materials, as in the present case, has not been reported yet. Such a steady tension in a uniform direction within a human tissue layer cannot be explained clearly by the previously described mechanisms. That migration might have occurred in superficial subcutaneous tissue layers through the horizontal flow or movement of those layers during the recovery process that have not been revealed yet.

**Electronic supplementary material:**

The online version of this article (doi:10.1186/1471-2482-14-103) contains supplementary material, which is available to authorized users.

## Background

A transverse suprapubic (Pfannenstiel) incision is widely used during cesarean section, and the skin closure is often performed with a subcuticular running suture, which might be associated with a low risk of wound complications compared to staples [1]. Either absorbable or nonabsorbable sutures can be used. Nonabsorbable sutures do not leave buried knots that can be associated with complications such as granulomas or abscesses [2]. However, the material of these sutures has a risk of incomplete removal after wound healing and can migrate to other sites in rare cases.

## Case presentation

**Figure 1:**
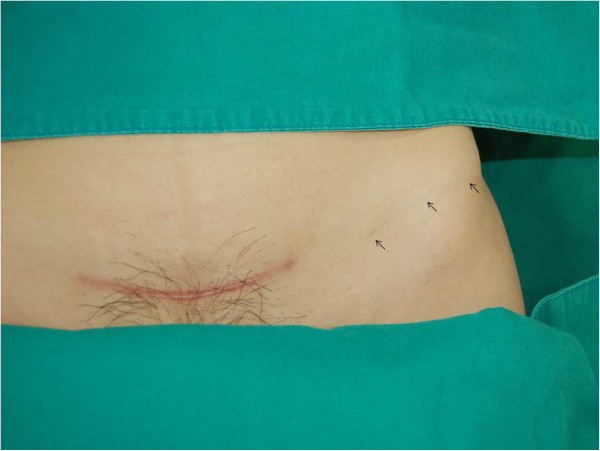
**The 10-cm thread-like object in the left lower quadrant of the abdomen (arrows).**

A 34-year-old, para 1, Korean woman underwent emergent cesarean section at 34 weeks of gestation in the Korea University Medical Center Anam Hospital in March 2012 because of preterm premature rupture of the membrane and breech presentation. Her body mass index (BMI) was 22.1 kg/m^2^. She was diagnosed with a uterus didelphys and vaginal septum but had no other medical or surgical history. A transverse suprapubic (Pfannenstiel) incision was performed during the cesarean section. A 2.42-kg male baby was delivered, without perioperative complications. After closure of the subcutaneous layer, the skin was closed with subcuticular running sutures by using a 3-0 nonabsorbable monofilament nylon suture (AILEE Inc., Busan, South Korea). The distal part of that string was snapped during the wound dressing on postoperative day 4, and the resident ordered the discharge of the patient on the next day without removal of the remaining string. Five months after the patient was discharged, she presented to the outpatient clinic with a fine, blue object visible on the left lower quadrant of her abdomen. A 10-cm thread-like foreign body showed through her skin. It was situated transversely; the medial end was located approximately 5 cm horizontally from the scar of the previous cesarean section, and the lateral end reached the upper lateral margin of the anterior superior iliac spine (Figures 1 [arrows] and 2). No pain or any other sign of inflammation was observed around the region. We discovered that the foreign body was the nonabsorbable suture material that had not been removed completely during the hospital stay. We thought that the remnant suture material had migrated laterally approximately 15 cm in dermis layer during the previous 5 months without any symptoms. Under local anesthesia with lidocaine, the material was removed easily through a 2-mm incision (Additional file 1: Video 1). The nylon suture material was confirmed, without the knot portion (Figure 3). The patient was discharged without any complications.

**Figure 2:**
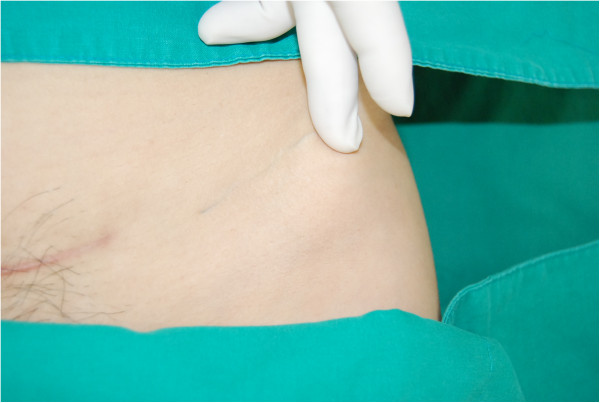
**The fine, blue foreign body showing through the skin.**

**Figure 3:**
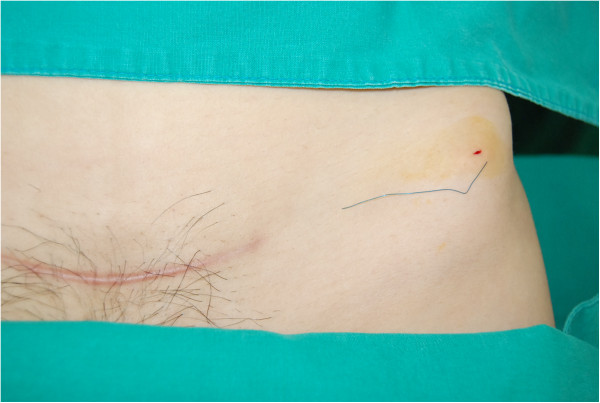
**The foreign body removed through a 2-mm skin incision.** The nylon suture material was confirmed, without the knot portion.

## Conclusions

Small remnants of nonabsorbable suture materials in the dermis or hypodermis (subcutaneous tissue) are known to migrate toward the superficial layer or occasionally to other sites within the human tissue. The phenomenon can be associated with complications, as reported in plastic surgeries such as blepharoptosis repair [3]. The mechanism of the migration was often thought to be related to foreign body immune reaction or to the force generated in wound contracture. However, no clear explanation has been proposed. To our knowledge, migration of relatively long suture materials, as in the present case, has not been reported yet. We retrieved related literature via PubMed by using keywords “migration”, “movement”, and “suture material”. In our case, the 10-cm length of the nylon suture migrated transversely to a relatively long distance (approximately 15 cm) within the dermis layer, without remarkable tangling of the entire length of the suture. Previously described mechanisms do not explain such uniform steady tension. Moreover, among suture materials, the nylon is known to have the least tissue reaction [4]. Some cases of migration of subcutaneous catheters, which are larger than suture materials, have been reported [5], but the mechanism of these migrations might be a shift in the subcutaneous fat pad due to position change, which is not applicable to the transverse migration of suture material.

Subcuticular skin sutures usually penetrate the dermis or superficial subcutaneous tissue. The subcutaneous tissue layer supports the mobility of dermal and epidermal skin layers [6]. The horizontal migration of the nonabsorbable suture in the present case might have occurred between the dermis and superficial subcutaneous tissue layers through the horizontal flow or movement of those layers, from the midline to the lateral side, during the recovery process or via mechanisms that have not been revealed yet.

More cases and studies are needed to explain the mechanism of the transverse migration of long suture materials. The results of these studies may provide evidence to better understand the movement of human tissues during wound recovery. Finally, practitioners need to be mindful that remnant suture materials can migrate to distant sites in rare cases. Careful exploration around the surgical wound might be helpful in these situations.

### Ethics

We obtained approval of this study from the institutional review board of the Korea University Anam Hospital Clinical Trial Center (reference No. 2014-05-0009).

### Consent

Written informed consent was obtained from the patient for the publication of this case report and any accompanying images. A copy of the written consent form is available for review by the editor of this journal.

## Electronic supplementary material

Additional file 1: Video 1: The migrated suture material identified and removed through a skin incision. A 10-cm-long, fine, blue material is seen transversely in the left lower quadrant of the abdomen; the medial end is located approximately 5 cm horizontally from the scar of the previous cesarean section, and the lateral end has reached the upper lateral margin of the anterior superior iliac spine. No sign of inflammation is observed around the region. Under local anesthesia with lidocaine, the material is removed easily through a 2-mm incision. (WMV 11 MB)
